# Mathematical Modeling of Amoxicillin Synthesis in Batch and Semi‐Batch Reactor: Application of Bayesian Statistics and Genetic Algorithm

**DOI:** 10.1002/bit.70096

**Published:** 2025-10-30

**Authors:** Lucas Figueiredo Formigosa, Ingrid Cabral dos Santos, Letícia Eduarda Alves e Álvares, Emanuel Negrão Macêdo, Luciana Rocha Barros Gonçalves, Bruno Marques Viegas

**Affiliations:** ^1^ Faculty of Biotechnology Federal University of Pará Belém Pará Brazil; ^2^ Graduate Program in Biotechnology Federal University of Pará Belém Pará Brazil; ^3^ Graduate Program in Chemical Engineering Federal University of Pará Belém Pará Brazil; ^4^ Department of Chemical Engineering Federal University of Ceará Fortaleza Ceará Brazil

**Keywords:** amoxicillin, genetic algorithm, kinetic parameters, Markov chain Monte Carlo method, modeling, penicillin G acylase

## Abstract

This study investigates the enzymatic synthesis of amoxicillin, focusing on its kinetic properties and their influence on antibiotic production in a batch‐operated enzymatic reactor. The reaction is catalyzed by penicillin G acylase (PGA, E.C.3.5.1.11), which is immobilized on glyoxyl‐agarose. The reaction involves the *p*‐hydroxyphenylglycyne methyl ester and 6‐aminopenicillanic acid (6‐APA) for amoxicillin formation. Under kinetic control, parallel hydrolytic pathways lead to product loss. Two kinetic models were evaluated: one based on Michaelis–Menten kinetics and another incorporating reaction and equilibrium constants for the process steps. Parameter estimation for the models was performed at different concentrations using two mathematical approaches: the Markov chain Monte Carlo (MCMC) method, rooted in Bayesian statistics and characterized as nondeterministic, and genetic algorithm, an evolutionary computation method incorporating crossover, mutation, and selection operators. The relative root mean squared error (rRMSE) was selected as the metric for evaluating the predictive performance of the models. MCMC presented the best results for low ester concentrations, with rRMSE values ranging from 1.48% to 6.10% for the Michaelis–Menten‐based model. The mathematical model was validated using data from an enzymatic reactor operating in semi‐batch mode, demonstrating a satisfactory capacity to predict the system's dynamic behavior under this operational condition.

## Graphical abstract

Synthesis of amoxicillin: The reaction catalyzed by penicillin G acylase is investigated in an enzymatic reactor, considering both Michaelis–Menten and mechanism‐based models with reaction and equilibrium constants. Parameters are estimated via MCMC and Genetic Algorithm, with MCMC showing superior performance in validating the mathematical model in a semi‐batch reactor.

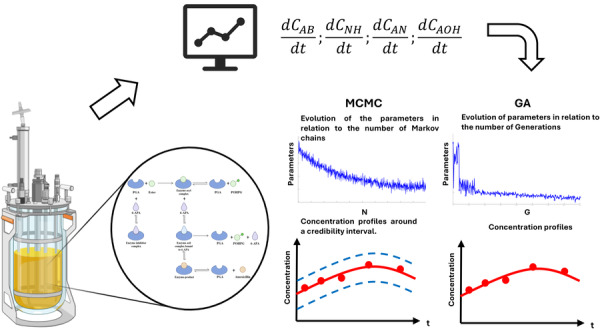

## 1 Introduction

Beta‐lactam antibiotics, known for their antibacterial properties, have significant commercial interest in various fields, including agriculture, medicine, and veterinary science (Cha and Carlson 2018). They are classified into different subgroups, including penicillins, cephalosporins, carbapenems, penems, and monobactams (Lima et al. 2020). Penicillins are considered the main beta‐lactam antibiotics due to their market value, which is estimated to reach USD 10.71 billion by 2024, encompassing natural, biosynthetic, and semi‐synthetic sources (Intelligence 2024). In the semi‐synthetic source, amoxicillin stands out due to its enhanced clinical properties, such as strong bactericidal activity, low toxicity, and broad spectrum of antimicrobial activities, driving interest in new sustainable synthesis approaches, particularly biotechnological ones (Tu et al. 2021).

The traditional production of semi‐synthetic beta‐lactam antibiotics relies on chemical routes that require specific operational conditions and toxic solvents, generating waste that is difficult to treat and compromising environmental sustainability (Pan et al. 2020). Due to the growing focus on climate change and stricter environmental protection laws (Paulus 2021; Chu et al. 2022), enzymatic synthesis emerges as an alternative, offering mild reaction conditions (25°C and pH 6.5), high selectivity, and a sustainable profile (Yan et al. 2023).

**Figure 1:**
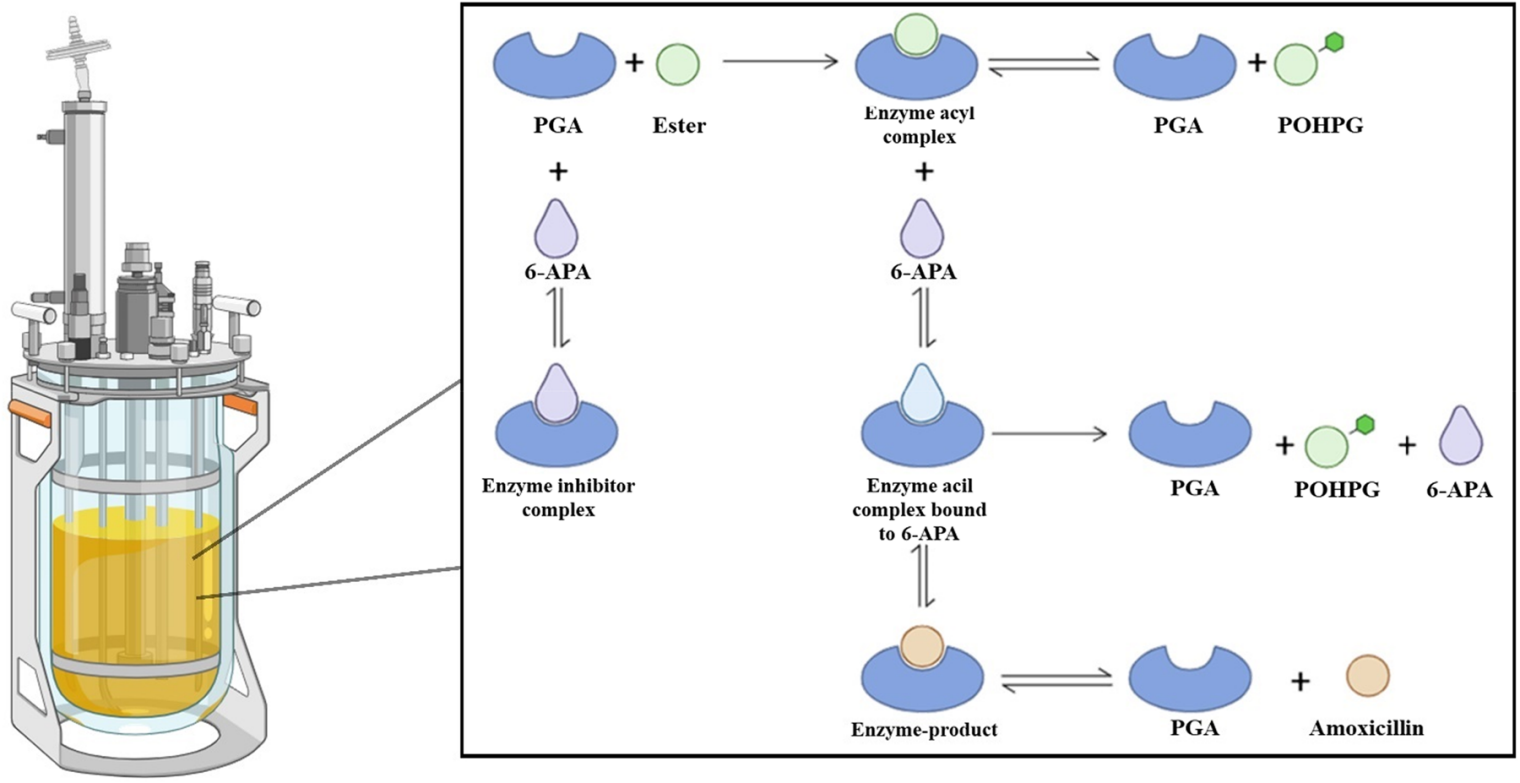
The amoxicillin synthesis process and its hydrolysis side reactions.

Sakaguchi and Murao (1950) identified Penicillin G Acylase (PGA), an enzyme from the fungus *Penicillium chrysogenum* capable of hydrolyzing penicillin G and biosynthesizing semi‐synthetic antibiotics (Pan et al. 2022). Since then, the most efficient enzyme for catalyzing the amoxicillin synthesis reaction is *Escherichia coli* Penicillin G Acylase (PGA, EC 3.5.1.11). Under kinetic control, the synthesis using PGA as a catalyst is a complex process. As illustrated in Figure 1, the reaction involves not only the formation of the desired product but also parallel hydrolysis processes. The *p*‐hydroxyphenylglycyne methyl ester (substrate) undergoes hydrolysis, as does the amoxicillin itself (product), generating p‐hydroxyphenylglycine (POHPG) as a byproduct. These hydrolytic processes significantly contribute to the reduction of the overall yield of the process (Pan et al. 2022; Yan et al. 2023).

Kinetic modeling of this process in a reactor is necessary to predict and design optimal configurations before the construction and industrial operation of the system. For the industry, kinetic models capable of representing a wide range of conditions relevant to large‐scale processes are essential (Lagerman et al. 2021). Despite the existence of several studies on enzymatic modeling and simulation in reactors (Gonçalves et al. 2000, 2003, 2005; Pan et al. 2020), there are still few that represent the complex enzymatic synthesis reactions of beta‐lactam antibiotics with multiple substrates and products, incorporating this wide range of operational conditions (Cuthbertson et al. 2019; Lagerman et al. 2021).

Bayesian statistics, based on Bayes' theorem, is a mathematical approach that defines a joint probability distribution for all parameters, both observable and unobservable, in a statistical model (Amador et al. 2022; Moura et al. 2022; Tavares et al. 2022; Cardoso et al. 2023; Soeiro et al. 2024). In this context, the Markov chain Monte Carlo (MCMC) method stands out as the most relevant approach for generating samples from arbitrary probability distributions. This technique has been widely applied in various processes, including the leaching of metal oxides from red mud, adsorption of pharmaceutical contaminants, production of 1,3‐propanediol by *Klebsiella pneumoniae* in batch bioreactors, and parameter estimation in population balances to describe particulate processes (Moura et al. 2021; Viegas et al. 2023; Dias et al. 2024; Moraes et al. 2025).

The genetic algorithm (GA) is an optimization technique based on the principles of natural selection, where each candidate solution is represented as a chromosome, whose properties can be mutated and recombined (Chen et al. 2024). This method has proven to be highly effective in solving multi‐objective problems and is widely applied in various fields of bioengineering, including the optimization of bioenergy production and wastewater treatment (Awhangbo et al. 2024; Chang et al. 2024; Elmalky and Araji 2024).

This study aims to select models and estimate the kinetic parameters of the amoxicillin synthesis reaction using penicillin G acylase (EC 3.5.1.11) in batch and semi‐continuous reactors at a temperature of 25°C and a pH of 6.5. Unlike previous studies, which mainly applied deterministic methods, the developed mathematical models were calibrated and validated using MCMC and GA methods, comparing them with experimental data from the literature (Gonçalves et al. 2000, 2003). The validation of the optimized parameters and the selected model was carried out through the synthesis reaction in a semi‐batch reactor, using exclusively parameters estimated in batch mode. This approach allowed the evaluation of the model's predictive performance, confirming its consistency and generalization. The quality of the fit was demonstrated by the low relative root mean squared error (rRMSE) values, evidencing the reliability of the obtained estimates.

## 2 Materials and Methods

### 2.1 Amoxicillin Synthesis

The experimental measurements were obtained by Gonçalves et al. (2000, 2003, 2005) using penicillin G acylase (PGA, EC 3.5.1.11) as the reaction biocatalyst. The PGA was immobilized in agarose gels through cross‐linking. The synthesis of amoxicillin was carried out in a 30‐mL batch reactor with mechanical stirring through the reaction between the methyl ester of p‐hydroxyphenylglycine and 6‐aminopenicillanic acid (6‐APA), catalyzed by penicillin G acylase immobilized on glyoxyl‐agarose. The other operating conditions considered for the batch and semi‐batch reactor for the enzymatic synthesis of amoxicillin are described in the experimental demonstration in the literature Gonçalves et al. (2000, 2003, 2005).

The experiments were conducted under two distinct initial concentration conditions: low concentration (80 mM ester and 60 mM 6‐APA) and high concentration (120 mM ester and 30 mM 6‐APA). The latter condition was established with the objective of developing a model capable of adequately representing the behavior of the system at high substrate concentrations (above 100 mM).

### 2.2 Mathematical Modeling

#### 2.2.1 Semi‐Empirical Kinetic Model (Model 1)

Model 1 was developed by Gonçalves et al. (2003), and it considers the following assumptions: (1) the antibiotic synthesis occurs after the binding of 6‐aminopenicillanic acid to the acyl‐enzyme complex; (2) the formation rate of the complex is not influenced by the presence of 6‐APA; and (3) the enzymatic reactor operates in batch mode under ideal mixing and agitation conditions.

Model 1 was based on Michaelis–Menten kinetics, enabling the determination of the substrate consumption rates (*v*_*AB*_), amoxicillin hydrolysis (*v*_*AN*_) and synthesis (*v*_*S*_). The dynamics of the process are described by a system of ordinary differential equations (ODEs), as presented in Equations ((1), (2), (3), (4)).

(1) $$\frac{dC_{AB}}{dt}=-v_{AB}=-\frac{K_{cat1}C_{AB}C_{EZ}}{K_{M1}(1+\frac{C_{AN}}{k_{AN}}+\frac{C_{AOH}}{k_{AOH}})+C_{AB}},$$

(2) $$\frac{dC_{AN}}{dt}=v_{S}-v_{AN}=v_{AB}T_{\max}\frac{C_{NH}}{K_{EN}+C_{NH}}-\frac{K_{cat2}C_{AN}C_{EZ}}{K_{M2}(1+\frac{C_{AB}}{k_{AB}}+\frac{C_{NH}}{k_{NH}}+\frac{C_{AOH}}{k_{AOH}})+C_{AN}},$$

(3) $$\frac{dC_{NH}}{dt}=v_{AN}-v_{S},$$

(4) $$\frac{dC_{AOH}}{dt}=v_{AB}-v_{S}+v_{AN}.$$

#### 2.2.2 Mechanism‐Based Model (Model 2)

Model 2 was developed by McDonald et al. (2019) and presents the following assumptions: (1) 6‐APA can inactivate the enzyme through direct binding to the active site; (2) the acyl‐enzyme complexes, both in the protonated and deprotonated states, can be inactivated when the enzyme is free; and (3) the enzymatic reactor also operates in batch mode under ideal mixing and agitation conditions (McDonald et al. 2019).

The rate equations allow the evaluation of the inhibitory effects of ester concentration (*C*_*AB*_), amoxicillin (*C*_*AN*_), 6‐APA (*C*_*NH*_) and the byproduct (*C*_*AOH*_). Model 2 was adapted to account for a constant enzyme concentration (*C*_*EZ*_), reflecting the experimental condition in which the enzyme was immobilized on a support. The four resulting ODEs define the production and consumption rates of the process, as described in Equations ((4), (5), (6), (7)).

(5) $$\frac{dC_{AN}}{dt}=R_{P}=\frac{C_{EZ}}{k_{3}K_{N}+k_{4}C_{NH}+k_{5}C_{NH}}\left(\frac{k_{2}k_{4}C_{AB}C_{NH}}{K_{S}}-\frac{k_{-4}C_{AN}\left(k_{3}K_{N}+k_{5}C_{NH}\right)}{K_{P}}\right),$$

(6) $$\frac{dC_{AOH}}{dt}=R_{B}=\frac{C_{EZ}\left(k_{3}K_{N}+k_{5}C_{NH}\right)}{k_{3}K_{N}+k_{4}C_{NH}+k_{5}C_{NH}}\left(\frac{k_{2}C_{AB}}{K_{S}}-\frac{k_{-4}C_{AN}}{K_{P}}\right),$$

(7) $$\frac{dC_{AB}}{dt}=R_{S}=-\left(R_{P}+R_{B}\right),$$

(8) $$\frac{dC_{NH}}{dt}=R_{NU}=-R_{P},$$

where *R_P_* represents the rate of production of amoxicillin, *R_B_* the rate of production of by‐product, *R_S_* the rate of consumption of the substrate (ester) and *R_NU_* is the rate of consumption of the β‐lactam nucleus.

#### 2.2.3 Semi‐Batch Model

The kinetic parameters previously estimated from batch experiments were used to validate the predictive capability of the model in amoxicillin synthesis under semi‐batch operating conditions. To represent this mode of operation, Models 1 and 2 were adapted with the inclusion of ester (*F*_*AB*_) and 6‐APA (*F*_*NH*_) feeding rates. The new ordinary differential equations that include these feed rates for model 1 are shown in Equations ((9), (10)) and for model 2 are Equations (11–12).

(9) $$\frac{dC_{AB}}{dt}=-V_{AB}+F_{AB},$$

(10) $$\frac{dC_{NH}}{dt}=V_{AN}-V_{S}+F_{NH},$$

(11) $$\frac{dC_{AB}}{dt}=R_{S}+F_{AB},$$

(12) $$\frac{dC_{NH}}{dt}=R_{NU}+F_{NH},$$

In this study, the inhibitory effects associated with both high and low substrate concentrations were investigated, as the literature shows limitations in several models when dealing with elevated concentrations. The estimated inhibitory kinetic parameters help to understand (i) how the products and byproducts influence the process yield at high concentrations; (ii) which species are considered inhibitory and remain present at high concentrations; and (iii) how the hydrolysis rates of the product and substrate are influenced across wide concentration ranges.

### 2.3 Optimization Algorithms

#### 2.3.1 Markov Chain Monte Carlo

Optimization algorithms for bioprocesses are of great importance for finding the optimal operating information, which can be defined even before the construction of the enzymatic reactor. For the application of optimization algorithms, the kinetic parameters estimated for both models were structured in the form of vectors, designated as P_1_ and P_2_, where index 1 corresponds to Model 1 and index 2 corresponds to Model 2. The state variables are represented by vector Y.

(13) $$P_{1}=\left[K_{cat1},K_{cat2},K_{M1},K_{M2},T_{\max},K_{EN},k_{AB},k_{AN},k_{AOH},k_{NH}\right],$$

(14) $$P_{2}=\left[k_{2},k_{3},k_{4},k_{-4},k_{5},K_{N},K_{S},K_{P}\right],$$

(15) $$Y=\left[C_{AB},C_{AN},C_{NH},C_{AOH}\right],$$

Bayesian statistics is a mathematical approach that integrates experimental information and a prior probability distribution to quantify the uncertainties associated with model parameters. This methodology generates a posterior probability distribution, allowing the analysis and estimation of parameters through the inverse formulation of the problem. The Bayes model is described by Equation (16) (Rényi 1955; Cardoso et al. 2023; Viegas et al. 2023).

(16) $$\pi (P|Y)=\frac{\pi (P)\pi (Y|P)}{\pi (Y)},$$

where vector P represents the model parameters, while Y corresponds to the vector of state variables. The posterior probability distribution is represented by π(P/Y), with π(Y/P) being the likelihood function and π(P) the prior probability distribution. The term π(Y) acts as a normalization constant. Thus, the posterior distribution is proportional to the product of the likelihood function and the prior distribution (Tavares et al. 2022; Viegas et al. 2023). In this study, the error standard deviation was fixed and no hyperparameters were estimated.

**Figure 2:**
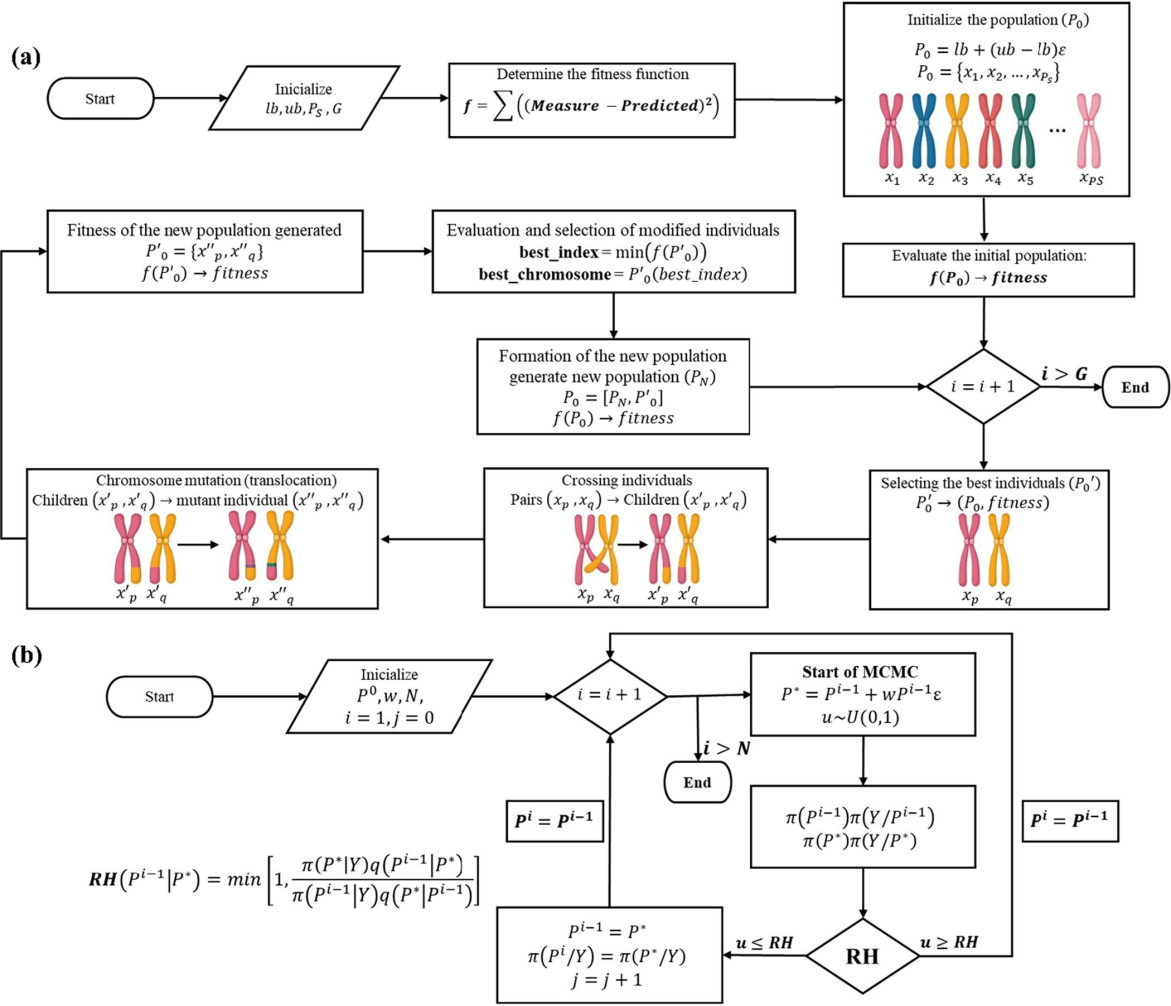
Flowchart of the estimation of kinetic parameters by (a) MCMC‐MH and (b) GA.

For the estimation of kinetic parameters, MCMC was used, considering a 99% credible interval (CI). The MCMC method was implemented in Matlab using the Metropolis‐Hastings (MH) algorithm, which aims to obtain random samples from an arbitrary probability distribution. Figure 2a shows the flowchart of the amoxicillin production process via enzymatic route, used for the determination of kinetic parameters in batch mode, through the MCMC‐MH method. This method selects a proposed distribution, which is a probabilistic function used to generate candidate parameter values for the next state of the Markov chain. This distribution is Gaussian in nature, characterized by generating candidates close to the current state of the chain based on a normal distribution, followed by an acceptance or rejection step of the suggested parameter values (Metropolis et al. 1953; Hastings 1970).

To implement MCMC using the MH algorithm, an initial parameter vector is first defined (**P** ^**0**^), The total number of iterations (N) and a search step (w) are defined. During the iterative process, the likelihood function is specified to calculate the posterior probability distribution. New candidates for the parameters (**P** ^*^) are generated from the parameter vector of the previous iteration (**P** ^**(i‐1** ***)***^), using a random term (ε) uniformly distributed as U(0,1). The acceptance or rejection of the new parameter vector is determined by the probability given by the Hastings Ratio (MH), as shown in the flowchart, Figure 2a. In this expression, *i* is the iteration counter, and *j* is the acceptance counter (Naveira‐Cotta et al. 2010; Tavares et al. 2022; Viegas et al. 2023).

A Gaussian prior probability distribution with a standard deviation of 0.6 was adopted, and the initial parameter values were defined based on references from the literature (Gonçalves et al. 2003). The algorithm was executed with 10^5^ iterations, using a relative step size of 6 × 10^−^^3^ and a deviation of 0.6 for the parameters. To ensure convergence of the chain to the equilibrium distribution, a burn‐in phase was established, after which the subsequent states were organized into a new parameter vector. From this vector, credible intervals were calculated using the quantile function in Matlab.

#### 2.3.2 Genetic Algorithm

GA is based on the principles of natural selection and genetics, where individuals better adapted to the environment have a higher probability of survival and reproduction. These algorithms operate on a population of candidate solutions, called individuals, which evolve over multiple generations. Each individual is represented by a chromosome, modeled as a parameter vector defining a feasible solution to the problem under analysis.

The process begins with the definition of the lower (**lb**) and upper (**ub**) bounds of the search space, the population size (*Ps*), and the number of generations (*G*). An initial population of possible solutions is randomly generated within these bounds, using the random variable ε. The performance of each solution is then evaluated through an objective function (fitness), which quantifies the deviation between the model's predictions and the experimental data.

The evolutionary process begins with the selection of the fittest individuals, using the roulette wheel method, where each solution has a selection probability proportional to its fitness index (Katoch et al. 2021). After selection, sparse crossover occurs, which combines the characteristics of the parent individuals to generate new offspring individuals, by creating a random binary vector that determines which characteristics will be inherited from each parent (Hakimi et al. 2016).

To maintain genetic diversity and avoid premature convergence to local minima, the mutation operator introduces random variations in the chromosomes of the new individuals (Katoch et al. 2021). This iterative cycle, consisting of selection, crossover, and mutation, is repeated until the stopping criterion is reached. The criterion is defined by the maximum number of generations *(i* > *G)*, where i represents the iteration counter of the algorithm.

This algorithm is presented in the flowchart of Figure 2b and was implemented in Matlab software. The operational parameters of the algorithm include a population size of 100, a total of 10^4^ generations, a crossover rate of 0.90, and a mutation rate of 0.10. The rRMSE was chosen as the metric for selecting the semiempirical models, as shown in Equation (17):

(17) $$rRMSE\equiv \frac{\sqrt{\frac{\sum_{i=1}^{n_{t}}{\left(Y_{m}-Y_{e}\right)}^{2}}{n_{t}}}}{{\bar{Y}}_{m}},$$

where *Y_m_* is the experimentally measured value, *Y_e_* is the estimated value, ${\bar{Y}}_{m}$ is the average of the experimental value and *n_t_* is total number of experimental measurements (Zhou et al. 2021).

## 3 Results and Discussion

### 3.1 Model Selection

**Figure 3:**
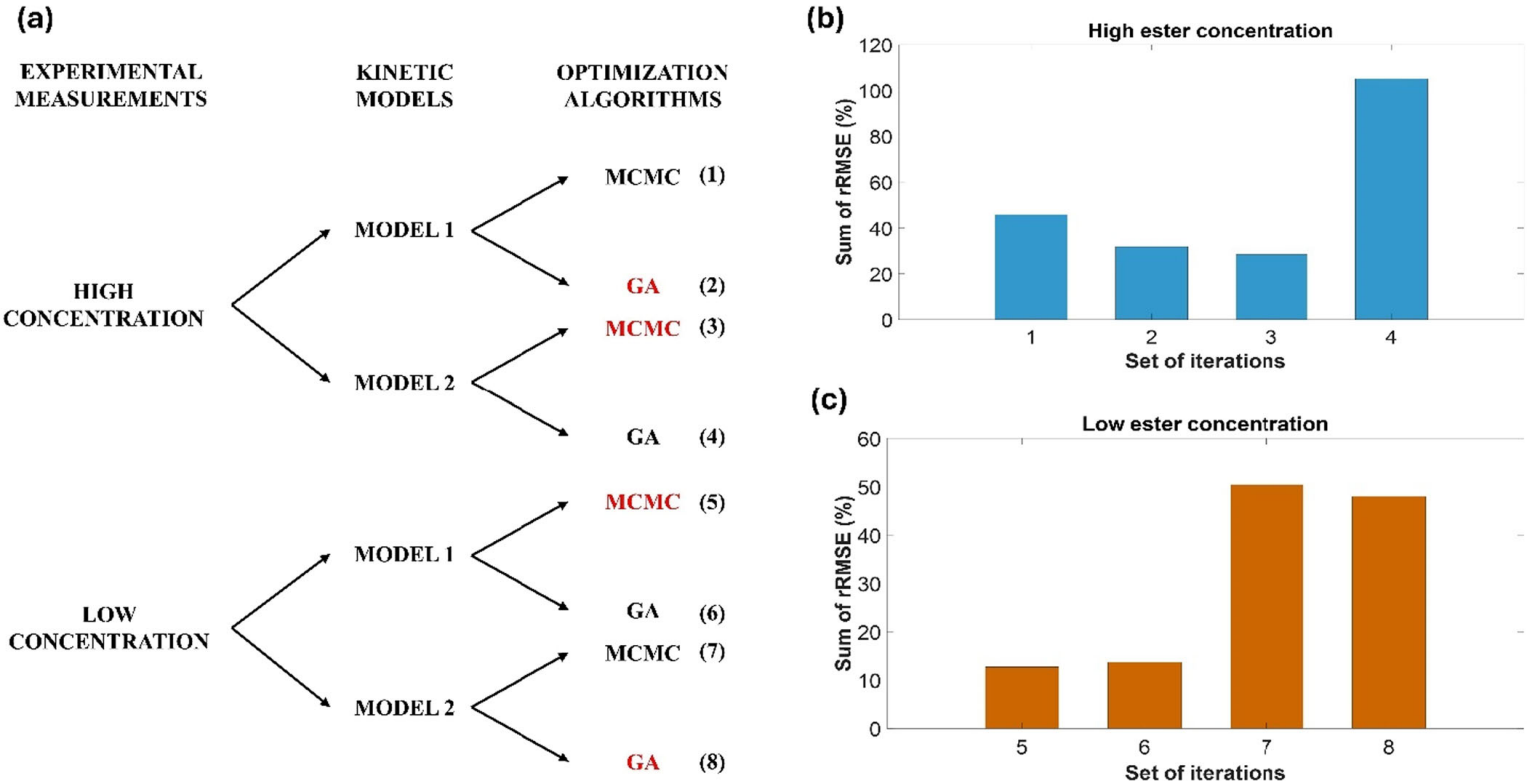
Overview of the analysis of (a) interactions between data, models, and optimization methods, (b) sum of rRMSE for high substrate concentration and (c) sum of rRMSE for low substrate concentration.

The objective of this study was to evaluate the interactions between parameter optimization techniques, mathematical models, and experimental data. The goal was to identify those interactions with the best performance, that is, the most accurate fit to the experimental data. These interactions are shown in Figure 3a. A total of eight interactions were conducted, with two selected for each of the proposed conditions (high and low), corresponding to one interaction for each model analyzed (model 1 and model 2) that demonstrated the optimal fit to the experimental data in each case. The histograms depicting the sum of the rRMSE of the high and low substrate concentrations are presented in Figure 3b,c, respectively.

### 3.2 Low Ester Concentration

In Table 1, for low ester concentration and using the MCMC method with Model 1, the catalytic constants (*K*_*cat*_) were analyzed. This constant represents the rate of conversion of the enzyme‐substrate complex into product and free enzyme (Michaelis and Menten 1913). The parameter *K*_*cat1*_ remained close to the initial estimate, with values of 0.18 µmol/i.u. min for MCMC. Meanwhile, *K*_*cat2*_, which considers the enzyme interaction with amoxicillin, showed a rate constant of 0.39 µmol/i.u. min for MCMC. These values suggest that the turnover rate is higher for amoxicillin compared to the ester.

**Table 1:** Estimating parameters in a batch reactor.

Parameters	Unit	Gonçalves et al. (2003)	Model 1		
			Low concentration ‐ MCMC	High concentration ‐ GA	
			Mean	99% CI	Value
*K*_*cat1*_	(μmol/i.u min)	0.18	0.181	(0.153; 0.234)	1.159
*K*_*cat2*_	(μmol/i.u min)	0.33	0.39	(0.279; 0.656)	1.785
*K*_*M1*_	(mM)	7.91	5.45	(4.113; 6.939)	43.48
*K*_*M2*_	(mM)	12.5	1.69	(1.43; 2.05)	49.22
*T*_*MAX*_	—	0.61	0.82	(0.71; 0.96)	0.98
*K*_*EN*_	(mM)	14.4	7.94	(5.65; 10.41)	32.48
*k*_*AB*_	(mM)	3.78	0.68	(0.43; 1.07)	645.03
*k*_*AN*_	(mM)	9.17	1.98	(1.17; 3.28)	815.80
*k*_*AOH*_	(mM)	10.9	9.85	(6.28; 13.26)	21.21
*k*_*NH*_	(mM)	62.04	9.76	(6.69; 14.47)	273.67

Parameters	Unit	McDonald et al. (2019)^a^	Model 2		
			Low concentration ‐ GA	High concentration‐ MCMC	
			Value	Mean	Value
*k*_*2*_	(min^−^^1^)	162^b^	0.029	0.027	(0.024; 0.033)
*k*_*3*_	(min^−^^1^)	44^b^	14.65	0.959	(0.61; 1.65)
*k*_*4*_	(min^−^^1^)	235^b^	3.90	0.066	(0.051; 0.084)
*k*_*5*_	(min^−^^1^)	9.0^b^	0.0014	0.152	(0.123; 0.211)
*K*_*S*_	(mM)	0.38^c^	13.29	3.193	(2.592; 3.894)
*K*_*P*_	(mM)	0.095^c^	1.89	0.239	(0.19; 0.30)
*K*_*N*_	(mM)	0.043^c^	19.37	1.655	(1.067; 2.471)
*k*_*‐4*_	(min^−^^1^)	217^b^	0.0033	0.003	(0.002; 0.0039)

^a^ Represents estimates of ampicillin synthesis.

^b^ Represents s^−^^1^.

^c^ Represents M (mol/L).

The affinity constant (*K*_*M*_) describes the interaction between PGA and the substrate, with *K*_*M1*_ referring to the ester and *K*_*M2*_ to amoxicillin. For *K*_*M1*_, the average estimate obtained was 5.45 mM, while for *K*_*M2*_ it was 1.69 mM. These values indicate that amoxicillin has a higher affinity for PGA compared to the ester. This behavior is explained by the role of amoxicillin as an intermediate product in the reaction. As the chemical equilibrium is reached, the hydrolysis of the antibiotic approaches its total conversion.

Table 2 presents the rRMSE values for the low ester concentration (LC) condition. Model 1 showed values between 1.48% and 6.10%, while Model 2 exhibited a wider error range, varying from 2.26% to 20.53% for the analyzed state variables. One hypothesis for the observed difference is that Model 2 does not account for the inhibitory effects caused by the reaction products and by‐products. Parameters such as product inhibition (*k*_*AN*_) and by‐product inhibition (*k*_*AOH*_) can significantly influence the nucleophilic attacks in the model and consequently affect the overall reaction kinetics at certain concentrations.

**Table 2:** Root‐mean‐square error (rRMSE) for concentrations.

Variables	rRMSE (%)			
	Model 1		Model 2	
	LC‐MCMC	HC‐GA	LC‐GA	HC‐MCMC
$C_{AB}$	2.39	4.50	2.26	9.23
$C_{AN}$	6.10	18.25	20.53	8.30
$C_{NH}$	1.48	4.16	5.37	4.08
$C_{AOH}$	2.75	4.83	19.87	6.84

The difference observed in the error values when comparing the two estimation methods may be explained by their distinct nature. MCMC performs stochastic sampling, exploring different regions of the parameter space according to the posterior probability distribution (Moraes et al. 2025). On the other hand, GA, although based on evolutionary processes, relies on well‐defined operators of selection, crossover, and mutation, which makes its exploration less random and more directed by deterministic criteria (Alhijawi and Awajan 2024).

In conditions of low ester concentration, Model 1 demonstrated superior performance, thereby evidencing its capacity to accurately represent the experimental data. This outcome can be ascribed to the incorporation of all inhibition constants within the model, a feature that enabled it to adequately capture the effects of competitive inhibition by products such as amoxicillin and by‐products. These by‐products assume greater significance at reduced substrate concentrations. This approach underscores the suitability of Model 1 for scenarios characterized by limited substrate availability, thereby reinforcing its applicability in these specific conditions.

**Figure 4:**
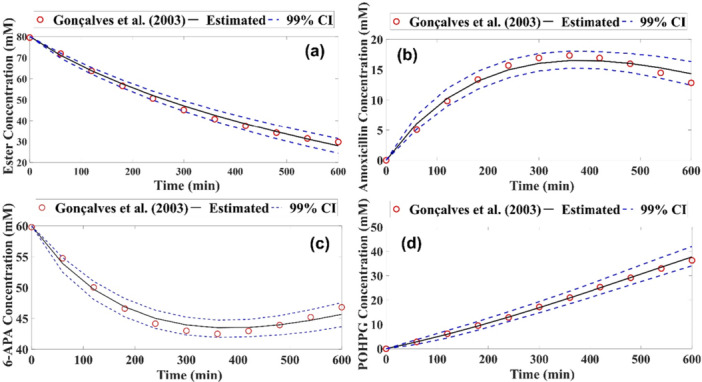
Experimental and estimated measurements of amoxicillin production at low ester concentration for Model 1 (a) Ester concentration (mM), (b) Amoxicillin concentration (mM), (c) 6‐APA concentration (mM) and (d) POHPG concentration (mM).

The results are shown graphically in Figure 4, representing interaction 5, Figure 3a. The experimental points are indicated by red circles, the solid line represents the average of the values predicted by the model and the blue dashed lines delimit the credible interval. All the experimental points are within this range with a 99% credible interval.

### 3.3 High Ester Concentration

The analysis of high substrate concentration in Model 2, using the MCMC‐MH method, suggests that the kinetic constant *k*_*3*_, related to the primary hydrolysis of the ester, is 0.95 (0.61; 1.65) min^−^^1^. In contrast, the constant *k*_*4*_, associated with the antibiotic synthesis reaction, has a value of 0.06 (0.05; 0.08) min^−^^1^. This difference between the constants (*k*_*3*_ **>** *k*_*4*_) indicates that hydrolysis is kinetically favored in the reaction, confirming the dynamics observed at low ester concentrations.

The dissociation constant for the enzyme‐product complex (*K*_*P*_) is 0.23 (0.19; 0.30) mM, suggesting that the product may have a significant affinity for the enzyme, leading to product inhibition. This implies that the product stays bound to the enzyme's active site for a longer period, potentially reducing the enzyme's availability to catalyze the synthesis reaction.

For Model 1, using the Genetic Algorithm, the estimated values for the parameter *K*_*cat1*_ were 1.16 µmol/i.u. min, indicating a higher conversion rate of the enzyme‐substrate complex into product. For *K*_*cat2*_, the value obtained was 1.78 µmol/i.u. min, confirming that the reaction rates are considerably higher compared to those observed at lower concentrations. However, the parameters maintain the same relationship (*K*_*cat2*_ *> K*_*cat1*_), indicating that the interaction between PGA and amoxicillin occurs more efficiently, requiring less time to complete the catalytic cycle.

Another important parameter is *T*_*max*_, which represents the conversion rate of the acyl‐enzyme complex into product. In the estimate proposed by the GA, the estimated value was 0.98, close to 1, indicating that almost the entire complex is converted into amoxicillin. However, based on previous studies (Ospina et al. 1996), it is known that nucleophilic attacks by water on enzyme complexes are frequent, which could reduce this conversion. A possible explanation for the high *T*_*max*_ value is the occurrence of a crystallization phenomenon during the reaction, which is recognized as one of the main causes of reactor clogging (Fan et al. 2024).

**Figure 5:**
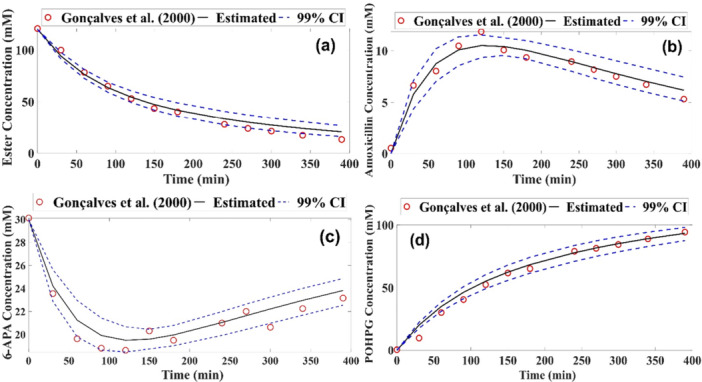
Experimental and estimated measurements of amoxicillin production at high ester concentration for Model 2 (a) ester concentration (mM); (b) amoxicillin concentration (mM); (c) 6‐APA concentration (mM); (d) by‐product concentration (mM).

In the high ester concentration (HC) condition, Model 1 showed rRMSE values between 4.16% and 18.25% with GA, while Model 2, using MCMC, presented errors ranging from 4.08% to 8.30%. The latter demonstrates excellent performance, as all state variables had errors below 10% (Zhou et al. 2021). The ensuing results are presented in graphical form in Figure 5, which illustrates the four profiles at a high concentration, thereby representing interaction 3 (Figure 3a).

In circumstances characterized by elevated ester concentrations, Model 2 demonstrated superior performance, even in the absence of explicit consideration for inhibition constants. This phenomenon can be attributed to the concept of competitive inhibition, wherein the presence of excess substrate displaces the inhibitor, thereby enabling the system to attain a consistent *Vmax* value. Consequently, the exclusion of inhibition effects from the model did not substantially compromise its capacity to align the experimental data at elevated substrate concentrations.

All simulations were performed on a computer with an Intel Core i5‐1135G7 processor (11th generation, 2.40 GHz), 16 GB of RAM, and Windows 10 Pro (version 22H2) operating system. The execution time for each interaction was approximately 2.33 h for GA and 2.28 h for MCMC.

### 3.4 Validation of the Mathematical Model

The estimated parameters were validated using experimental data obtained in a semi‐batch reactor, maintaining a constant enzyme concentration. In this approach, the substrates (ester and 6‐APA) were added in solid form. Considering that the substrate concentration in the semi‐batch reactor was relatively low (6 g/L), the application of Model 1 using the MCMC method was chosen.

The tested models were calibrated based on experimental data obtained in batch mode and subsequently applied to predict the system's behavior under semi‐batch conditions. This approach allowed for the evaluation of the consistency and applicability of the estimated kinetic parameters, considering that the nature of the reaction remains unchanged regardless of the reactor's operational conditions.

**Figure 6:**
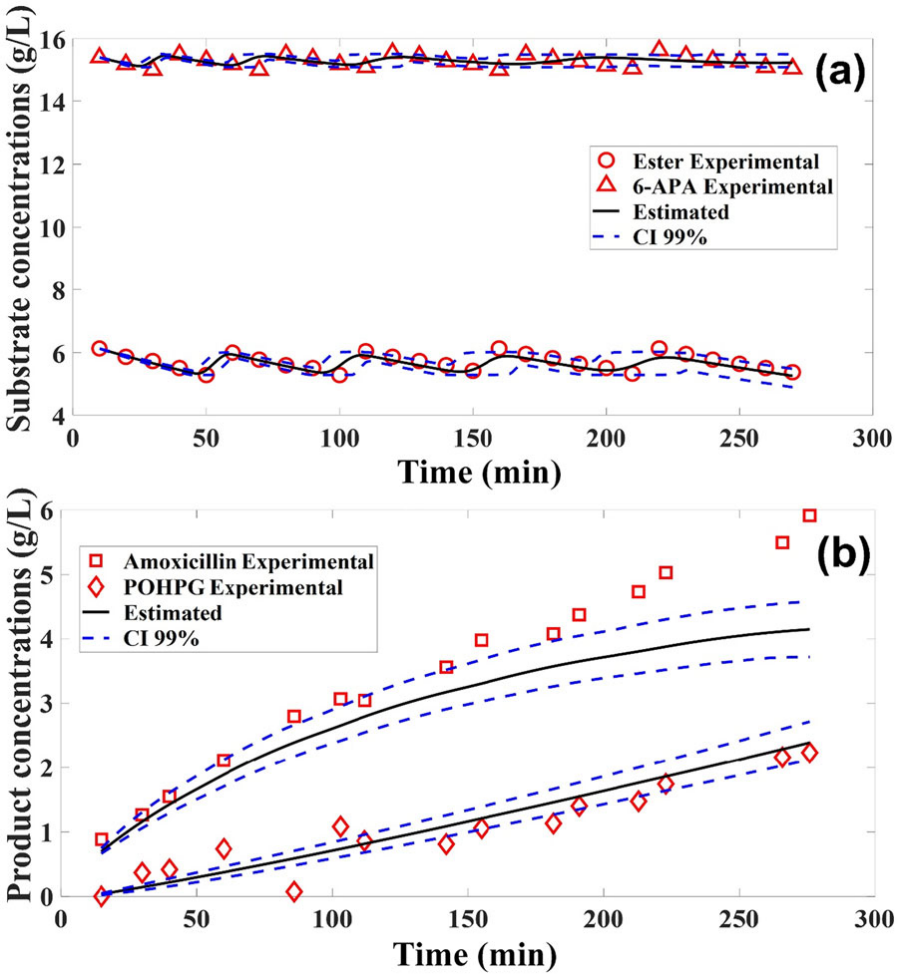
Validation of the mathematical model using Model 1 at low ester concentration (a) concentrations of ester and amoxicillin (g/L), (b) concentrations of the products formed, amoxicillin and POHPG (g/L).

The predictions of substrate and byproduct concentrations showed good agreement with the experimental data (Figure 6), with the measurements within the credible interval. However, for the product concentration profile, the model underestimated amoxicillin formation after approximately 200 min, suggesting a potential inhibitory effect resulting from the byproduct concentration. The validation of the analyzed concentrations reinforces the effectiveness of the model in representing the overall system dynamics, suggesting that it can provide reliable predictions under different operating conditions.

## 4 Conclusion

The findings indicated that Model 1, employing the MCMC method, exhibited superior performance at low ester concentrations, with rRMSE values ranging from 1.48% to 6.10%. This enhanced performance was ascribed to the incorporation of competitive inhibitors, such as amoxicillin and by‐products, into the consumption and hydrolysis of the ester, thereby ensuring a more precise fit to the experimental data. This finding underscores the significance of incorporating the effects of competitive inhibition in scenarios where substrate availability is limited.

Model 2 stood out at high ester concentrations, with errors ranging from 4.08% to 8.30%, demonstrating its ability to capture the dynamics of the system in these cases. This model benefited from the use of reaction and equilibrium constants, including consideration of active site inhibition by 6‐APA. When comparing the methods of adaptation, MCMC outperformed GA in terms of both predictive power and experimental validation. However, improvements to GA, such as adjustments to the crossover, mutation and selection phases, and expansion of the search space, could improve its effectiveness in the synthesis of amoxicillin.

The validation of the models and methods reinforces their applicability in different scenarios, from batch to semi‐batch processes, highlighting the relevance of MCMC in enzyme synthesis. The results provide a promising outlook for future improvements, such as the inclusion of additional phenomena, such as crystallization, to make the modeling even more robust. This study provides a solid basis for further studies in mathematical modeling and for the development of more precise and efficient industrial enzymatic processes.

## Nomenclature and Symbols

### 1 Nomenclature

6‐APA: 6‐aminopenicillanic acid
Ester: *p*‐hydroxyphenylglycyne methyl ester
GA: Genetic Algorithm
HC: High concentration
LC: Low concentration
MCMC: Markov chain Monte Carlo
MH: Metropolis‐Hastings Algorithm
POHPG: *p*‐hydroxyphenylglycyne
rRMSE: Relative root mean squared error

### 2 Symbols

K_cat1_
: catalytic constant of the enzyme in relation to the substrate (μmol/i.u min)
f
: fitness function
G
: number of generations
$k_{2}$
: formation constant of the acyl‐enzyme complex (min^‐1^)
$k_{3}$
: constant for the hydrolysis reaction of the ester (min^‐1^)
$k_{4}$
: kinetic constant associated with the product synthesis step (min^‐1^)
$k_{‐4}$
: constant represents the speed of the reverse reaction of the step associated with $k_{4}$(min^‐1^)
$k_{5}$
: kinetic constant related to the formation of by‐products by the product formation route (min^‐1^)
$k_{\text{AB}}$
: inhibition constant of the ester (mM)
$k_{\text{AN}}$
: inhibition constant of the product (mM)
$k_{\text{AOH}}$
: inhibition constant of the byproduct (mM)
$K_{\text{cat2}}$
: catalytic constant of the enzyme in relation to the product (μmol/i.u min)
$K_{\text{EN}}$
: adsorption constant for 6‐APA (mM)
$K_{\text{M1}}$
: Michaelis–Menten constants for ester consumption (mM)
$K_{\text{M2}}$
: Michaelis–Menten constants for the hydrolysis of amoxicillin (mM)
$K_{N}$
: nucleophile association constant (mM)
$k_{\text{NH}}$
: inhibition constant of the β‐lactam core (mM)
$K_{P}$
: equilibrium constant associated with product formation (mM)
$K_{S}$
: equilibrium constant relating enzyme and substrate interaction (mM)
lb
: lower parameter value limit
N
: number of states in the Markov chain
$n_{t}$
: total number of experimental measurements
$P^{*}$
: vector of candidate parameters of the Monte Carlo method via Markov chain
$P^{0}$
: vector of initial estimates parameter
$P_{0}$
: initial population
$P_{0}^{\prime }$
: selecting the best individuals
$P_{N}$
: new population
$P_{S}$
: population size
t
: time (t)
$T_{\mathrm{MAX}}$
: maximum conversion ratio of the acyl‐enzyme‐core complex into product (mm)
ub
: upper parameter value limit
$V_{MAX}$
: maximum reaction velocity
w
: relative standard deviation
x
: individual or chromosome
Y
: vector of state variables of the mathematical model
$Y_{e}$
: vector of estimated parameters
$Y_{m}$
: vector of experimental measurements
${\bar{Y}}_{m}$
: vector of the means of experimental measurements

### 3 Greek Symbols

ξ
: random variable U (0,1)
π(P)
: prior probability distribution
π(Y)
: marginal probability density of measurements
π(P|Y)
: posterior probability distribution
π(Y|P)
: likelihood function

## Author Contributions

**Lucas Figueiredo Formigosa:** methodology, software, validation, visualization, writing‐original draft. **Ingrid Cabral dos Santos:** formal analysis, writing – review and editing. **Letícia Eduarda Alves e Álvares:** formal analysis, writing – review and editing. **Emanuel Negrão Macêdo:** software, validation, writing – review and editing. **Luciana Rocha Barros Gonçalves:** conceptualization, methodology, software, validation, writing – review and editing, supervision. **Bruno Marques Viegas:** conceptualization, methodology, software, validation, writing – review and editing, supervision.

## Conflicts of Interest

There is no relationship with any person or organization that affected the results and conclusions of this study.

## Data Availability

The data that support the findings of this study are available from the corresponding author upon reasonable request.
